# The Varieties of Lymphangitis

**Published:** 1891-11-07

**Authors:** 


### SURGERY.

### THE VARIETIES OF LYMPHANGITIS.

The important part played by the lymphatics in absorption renders them liable to many kinds of infection. Fortunately, however, they are not very susceptible to inflammation, and irritating and poisonous substances, both from free surfaces, such as the skin and mucous membrane, and from the disintegration of the tissues, pass through them without harm. It is probable that the lymph corpuscles contained in the simple and compound lymphatic glands have the property of destroying or altering both germs and other noxious matter. Hence, it is still a doubtful point whether there is such a thing as "idiopathic lymphangitis"; in other words, it seems to usually require a very definite poison or injury to induce inflammation in these vessels. The term "idiopathic," therefore, if retained at all, must be applied to those cases where there is no *obvious* cause for the disease.

In an article on this subject in *L'Union Medicale* for October 1st, 1891, M. Morel-Lavallée describes the following varieties:—

1. Lymphangitis of the Trunks.—This is rarely seen in a pure state except in torpid indurated conditions, as after the absorption of syphilis.

2. Non-infectious Capillary Lymphangitis. — This usually precedes and accompanies the above. It differs from the next variety only in its want of imparting itself when inoculated. But this is not always the case; hence it is nearly allied to

3. Cutaneous Erysipelas.—The actual specific nature of this is by no means certain. M. Clado, working in Professor Verneuil's laboratory, has shown that the microbe of an ordinary lymphatic abscess will produce the same result when inoculated on the rabbit as the streptococcus of erysipelas. It should be remembered, nevertheless, that the course of this disease is clinically so distinct, that it is rather to be expected that its pathology will be ultimately found to be equally definite.

4. Lymphangitis with Nodular Enlargement.—This is diagnosed by the varicose condition of the vessels, easily felt by the finger. These "knots" are probably (as Bazin points out) due to the obstruction and increased inflammation at the valves. Hence, the latter observer calls it "valvular."

5. Ampullary lymphangitis.—This resembles the last variety in many respects, but the small lumps are more definitely formed, and are not apparently confined to the neighbourhood of the valves. They may occur also with or without the intervening trunks being obviously affected. Cases of this variety have been recorded by Merklen, Hollopean, and Fournier, &c., but in England the disease has been recognised under a different name, namely, "tuberculosis of the skin." This does not, of course, include lupus, or the deposit of so-called "tubercle," irrespective of the lymphatics. The nodules are usually adherent to the skin, but sometimes free from it, and are arranged in vertical series, as interrupted cords. They vary in size from a minute speck to that of a mandarin orange. Occasionally they suppurate, or even ulcerate.

It must be confessed that this condition as described by French surgeons is very rare, but in a less pronounced form, with a few nodules running in the course of the lymphatics in a tuberculous subject it is more often (though rarely) seen.

An interesting point has been brought out by some experiments of M. Felizet. He injected some tincture of iodine (20 to 30 drops) into tubercular glands in the neck, and found that the pain that followed did not occur at the seat of puncture, but lower down, evidently in the next glands. In another case in which the cervical glands were enlarged and there was difficulty of breathing in a child aged nine years, he followed out the same plan, injected the iodine into the uppermost cervical gland. The dyspnœa became rapidly worse, and then improved. After several injections, each followed by the same symptoms, the child was cured. Evidently the bronchial glands were enlarged, and pressed on the air tubes; they became inflamed and œdematous after the infection, but disappeared ultimately. In these cases there is evidence of the permeability of the lymphatics when they are in an advanced stage of disease. Even when the glandular affection has gone on to suppuration the injection of a solution of iodine in ether has permeated into neighbouring glands. This is an important point both in pathology and treatment. Amongst other lessons, it teaches how dangerous a caseating or suppurating gland may be as a centre for general infection, and how imperative it is to scrape or remove such whenever this is practicable.

